# α-Conotoxin Peptidomimetics: Probing the Minimal Binding Motif for Effective Analgesia

**DOI:** 10.3390/toxins12080505

**Published:** 2020-08-06

**Authors:** Adam C. Kennedy, Alessia Belgi, Benjamin W. Husselbee, David Spanswick, Raymond S. Norton, Andrea J. Robinson

**Affiliations:** 1 School of Chemistry, Monash University, Clayton, Victoria 3800, Australia; adam.kennedy@monash.edu (A.C.K.); alessia.belgi@monash.edu (A.B.); benjamin.husselbee@monash.edu (B.W.H.); 2 Biomedicine Discovery Institute and the Department of Physiology, Monash University, Victoria 3800, Australia; David.Spanswick@monash.edu; 3 Warwick Medical School, University of Warwick, Coventry CV4 7AL, UK; 4 NeuroSolutions Ltd., Coventry CV4 7AL, UK; 5 Medicinal Chemistry, Monash Institute of Pharmaceutical Science, Monash University, 381 Royal Parade, Parkville, Victoria 3052, Australia; ray.norton@monash.edu; 6 ARC Centre for Fragment-Based Design, Monash University, Parkville, Victoria 3052, Australia

**Keywords:** conotoxins, peptides, analgesia, disulfide, dicarba peptides, GABA_B_, nAChR.

## Abstract

Several analgesic α-conotoxins have been isolated from marine cone snails. Structural modification of native peptides has provided potent and selective analogues for two of its known biological targets—nicotinic acetylcholine and γ-aminobutyric acid (GABA) G protein-coupled (GABA_B_) receptors. Both of these molecular targets are implicated in pain pathways. Despite their small size, an incomplete understanding of the structure-activity relationship of α-conotoxins at each of these targets has hampered the development of therapeutic leads. This review scrutinises the *N*-terminal domain of the α-conotoxin family of peptides, a region defined by an invariant disulfide bridge, a turn-inducing proline residue and multiple polar sidechain residues, and focusses on structural features that provide analgesia through inhibition of high-voltage-activated Ca^2+^ channels. Elucidating the bioactive conformation of this region of these peptides may hold the key to discovering potent drugs for the unmet management of debilitating chronic pain associated with a wide range of medical conditions.

## 1. Introduction

Venomous marine cone snails of the genus *Conus*, estimated to contain more than 700 species, possess a unique mixture of pharmacologically-active peptides [1,2,3,4]. Conotoxins are found in the venom duct and comprise a large family of small disulfide-rich peptides that typically contain 12–30 amino acids [5]. These natural products have been broadly categorised based on their gene superfamily, and their highly-conserved disulfide connectivity [6]. The high structural diversity of conotoxins provides exquisite selectivity at a range of mammalian ion channels and receptors, including nicotinic acetylcholine receptors (nAChRs) and sodium, potassium and calcium ion channels [7].

α-Conotoxins were among the first-discovered peptides in the conotoxin family and were initially shown to target nAChRs. They are typically shorter than other conotoxins, with only 12–20 amino acids, contain a highly-conserved Cys I-III, II-IV globular disulfide framework, and possess a well-defined three-dimensional structure [8]. In combination with a conserved turn-inducing proline residue, the interlocked disulfide bridge framework stabilises the three-dimensional architecture into two clear domains—Loop I (*N*-terminus to residue 8) and Loop II (residue 9 to the *C*-terminus). This stabilised structure results in peptides that typically display high efficacy, potency and selectivity for their receptor targets, making them prime candidates for drug development [9,10]. Indeed, several have been proposed as suitable candidates in the treatment of many diseases, including Alzheimer’s disease, Parkinson’s disease, epilepsy, cardiac infarction, hypertension and neuropathic and chronic pain [3,10,11,12,13,14]. To date, however, no α-conotoxins are in clinical use.

**Figure 1:**
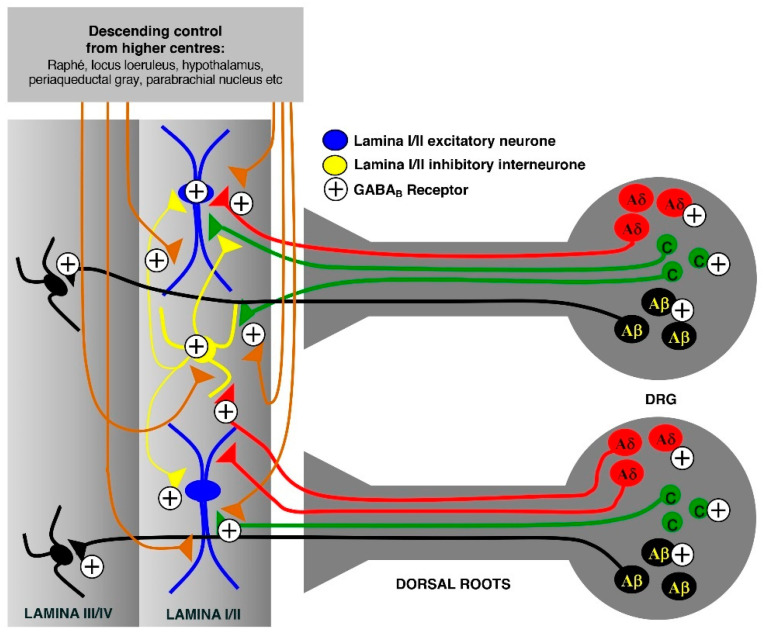
γ-Aminobutyric acid G protein-coupled (GABA_B_) receptor distribution and overview of pain signalling in the dorsal horn via modulation from higher centres.

This review focuses on analgesic α-conotoxins. The best-characterised targets for these peptides are nAChRs, where they display nanomolar potency and subtype specificity. Significantly, these receptors have been implicated in pain pathways [15]. However, recent evidence has also emerged that several α-conotoxins indirectly inhibit high-voltage activated (HVA) Ca^2+^ channels via agonism of γ-aminobutyric acid (GABA) G protein-coupled (GABA_B_) receptors, which are also implicated in pain transmission [16,17,18,19]. GABA_B_ receptors are widely expressed and distributed in pain-processing pathways at all levels of the neuraxis and play an extensive role in editing and modulating nociceptive inputs [20,21,22,23,24]. This occurs at peripheral dorsal root ganglia (DRG) and first-order neurons, and at the level of the dorsal horn of the spinal cord where first-order neurons engage the central neural circuits (Figure 1). GABA_B_ receptors are located at both pre- and postsynaptic sites and, at the level of the DRG, are expressed in large (Aβ) [25], medium (Aδ) and small diameter (C-fibre) neurons [26]. At the level of the dorsal horn, GABA_B_ receptors are located on the presynaptic terminals of the primary afferents arising from the DRG and on cell bodies and processes of interneurones in laminae I, II and III, the latter involved in processing tactile sensory information [27]. They are also located on presynaptic terminals originating from descending inputs from higher centres. Presynaptic GABA_B_ receptors on the primary afferent terminals of Aδ and C fibres synapsing with lamina I and II dorsal horn neurones have an important role in modulating nociceptive transmission from the periphery, acting to suppress glutamate and peptide release from these terminals. Similarly, presynaptic GABA_B_ receptors inhibit GABA and glycine release from spinal inhibitory interneurones. While the target/s of analgesic α-conotoxin sequences is still under investigation, there is little doubt that they hold great promise for development as therapeutic agents. Additionally, sequence modification of native α-conotoxins provides analogues with improved plasma stability and altered receptor affinity and potency, as well as the opportunity to probe their structure-activity relationships.

Several native α-conotoxin sequences are known to be effective painkillers, inducing potent analgesia without the associated side effects of dependence and tolerance typically associated with opiates [28,29,30]. The unparalleled selectivity and potency of these peptides are highly desirable in pharmaceutical design, although short biological half-lives, low oral bioavailability and poor metabolic stability limit the potential of the unmodified peptides as drug candidates [31,32]. The challenge remains to retain or enhance the bioactive properties of an α-conotoxin peptide in drug-like molecules that can be administered clinically. Success in the pharmaceutical space has been achieved with Ziconotide (ω-conotoxin MVIIA), which is used to suppress pain through negative modulation of N-type calcium channels [28]. To date, Ziconotide is the only conotoxin available on the market, although several promising analgesic α-conotoxin lead sequences have been identified (Table 1).

## 2. Therapeutic α-Conotoxin Development 

**Figure 2:**
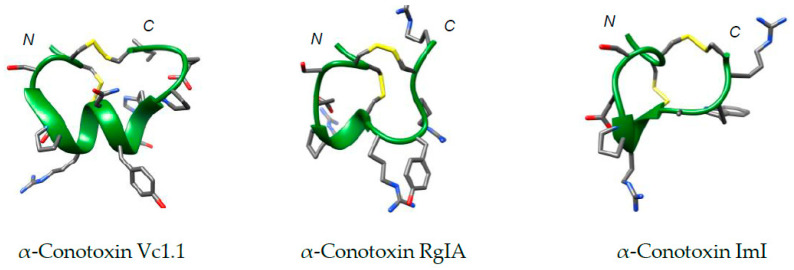
Structures of α-conotoxins Vc1.1 (PDB: 2H28), Rg1A (PDB: 2JUT), and ImI (PDB: 1IMI) calculated from solution-state NMR data, provided from the Protein Data Bank (PDB) [34,43,45]. Structures were produced using Chimera [46]. These peptides share identical Loop I residues (GCCSDPRC) and possess variable Loop II primary sequences (full sequences are shown in Table 1). Peptide backbone shown in green, disulfide linkages in yellow and *N*- and *C*-termini are labelled.

α-Conotoxin Vc1.1 was first identified in the venom of the cone snail *Conus victoriae* through cDNA screening in 2003 [33]. It contains 16 residues, an amidated *C*-terminus, two disulfide bonds with a I-III, II-IV (globular) connectivity and a central helical region spanning residues 6–11 (Figure 2). The cysteine spacing within its sequence defines it as a member of the 4/7 subclass of α-conotoxins, with four amino acids between the cysteine residues in Loop I and seven amino acids in Loop II [34,35]. Since the discovery of Vc1.1, its ability to alleviate pain in animals with peripheral neuropathy and to selectively inhibit neuronal nAChRs over muscular variants has made it a promising candidate for neuropathic pain treatment [33,35]. Analgesic efficacy was originally attributed to the inhibition of α9α10 nAChR [36]. Indeed, Vc1.1 was taken to clinical trials by Metabolic Pharmaceuticals but was later withdrawn due to dramatically-reduced potency at the human α9α10 nAChR compared with the equivalent rat receptor [37]. Thereafter, in vitro analyses of rodent dorsal root ganglia (DRG) identified that the analgesic effect of Vc1.1 could arise from indirect inhibition of HVA Ca^2+^ channels via GABA_B_ agonism [16,17,18,19]. Currently, the mechanism of analgesia is still highly contentious, and debate continues about the primary receptor/s responsible for analgesia.

In 2010, an *N*-to-*C* backbone cyclised analogue of Vc1.1, cVc1.1, was developed and shown to possess increased activity at the GABA_B_ receptor (IC_50_ = 0.3 nM vs. 1.7 nM for cVc1.1 and Vc1.1, respectively) but decreased inhibition of α9α10 nAChRs (IC_50_ = 766 vs. 64 nM for cVc1.1 and Vc1.1, respectively) [38]. Importantly, cVc1.1 also displayed high stability against proteolytic degradation in serum, simulated gastric fluid and simulated intestinal fluid [38], conferred in part by native Vc1.1′s exceptional stability in each of these models over 24 h. Significantly, both native Vc1.1 and cVc1.1 are able to alleviate mechanical allodynia after oral administration in a rodent model of neuropathic pain [38]. Additionally, both Vc1.1 and cVc1.1 are able to inhibit mouse colonic nociceptors via activation of GABA_B_ receptors, although cVc1.1 displayed a greater potency (IC_50_: 12.2 nM vs. 23.4 nM for native Vc1.1) [39,40], highlighting the potential for an α-conotoxin-derived peptide therapeutic.

Analgesic α-conotoxin RgIA was identified in 2006 via PCR amplification of the DNA of *Conus regius* and was classified as a novel α9α10 nAChR antagonist. It is a 13-residue peptide member of the 4/3 disulfide subclass and retains the conserved structural scaffold of α-conotoxins with a central helical region and residue 1–8 sequence homology with Vc1.1 (Table 1, Figure 2) [41,42,43]. As with Vc1.1, the inhibitory effect of RgIA at the α9α10 nAChR was considered to be the mechanism of analgesia in animal models of neuropathic pain [36,43]. However, it was later shown to also inhibit HVA Ca^2+^ channels via agonism of the GABA_B_ receptor (IC_50_ = 40.7 nM (rat)) [16,17,44]. In 2011, several backbone-cyclised analogues of RgIA, which have the same activity as RgIA but lack the terminal Arg13 residue, were synthesised and evaluated at both α9α10 nAChR and GABA_B_ receptors [44]. Interestingly, addition of a single residue to the cyclising linker sequence offered target selectivity, leading to increased inhibition of α9α10 nAChR but decreased activity at the GABA_B_ receptor [44].

In an attempt to address the confusion over the receptor pathways that mediate analgesia, an analogue containing several point mutations across Loops I and II (RgIA4) was developed [15]. RgIA4 was tested on mouse, rat and human α9α10 nAChR and showed low nanomolar affinity at each of these receptors. This analogue was able to prevent chemotherapy-induced cold allodynia up to 21 days after final administration, suggesting it could provide long-lasting protection from nerve damage [47]. These findings strongly support the involvement of α9α10 nAChRs in the expression of pain.

α-Conotoxin AuIB, identified from *Conus aulicus,* is a 15-residue peptide of the 4/6 disulfide subclass. It was initially identified as a selective antagonist of the α3β4 subtype of nAChR over α7 and α4β2 subtypes [48,49,50]. *N*-to-*C* backbone cyclisation of AuIB using a variety of spacer linkages improved serum stability compared to the native peptide and showed promise as a probe for the role of α3β4 nAChRs in in vivo pain models [48,49]. AuIB is also able to inhibit HVA Ca^2+^ channels at nanomolar concentrations via a GABA_B_-dependent mechanism (IC_50_ = 1.5 nM in rat DRG neurons) and modulates mechanical allodynia in the partial nerve ligation (PNL) model [51]. The wild-type globular disulfide connectivity is considered to be the more stable and active form of peptide structure in α-conotoxins, but AuIB was the first example of a nAChR subtype-selective ribbon topoisomer (Cys I-IV, II-III) displaying a higher potency than its globular counterpart [52]. A detailed structure-activity relationship study shed light on the residues of ribbon AuIB crucial for its activity and established the role of ribbon isomers of α-conotoxins as molecular probes for specific subtypes of nAChRs [53].

PeIA, from *Conus pergrandis*, is another α-conotoxin member of the 4/7 subclass [54,55], and a selective inhibitor of α9α10 nAChR with a similar secondary structure to that of Vc1.1 (determined by NMR spectroscopy) [54]. Owing to its similarity to Vc1.1 and its potent inhibition of α9α10 nAChR, PeIA was also tested for inhibition of HVA Ca^2+^ channels via GABA_B_ receptor agonism and found to be active at nanomolar concentrations (IC_50_ = 1.1 nM in rat DRG neurons) [54]. 

Recently, an alternative strategy to improve the potency of α-conotoxins toward α9α10 nAChR by formation of dimeric peptides was reported [56]. α9α10 nAChR expressed from a high ratio of α9 and α10 mRNA contained two neighbouring binding sites for α-conotoxins that could be concomitantly targeted by a single dimeric conotoxin sequence and therefore improve binding affinity. Three α-conotoxins, Vc1.1, PeIA and [*des*-R13]-RgIA-NH_2_, were dimerised using solution phase copper-catalysed azide-alkyne cycloaddition (CuAAC) click chemistry between sequence-installed *N*-terminal azido- and alkyne-derivatised lysine residues. Dimers of Vc1.1, [*des*-R13]-RgIA analogues, and PeIA showed concentration-dependent inhibition of human α9α10 nAChR with potencies increased by ~4-, 7- and 11-fold over native values, respectively. The dimers also displayed interesting activity at human α7 nAChR, a sub-type recently shown to be over-expressed in several types of cancer together with the α9 subunit [56,57]. 

The sequence of α-conotoxin Pu1.2 from *Conus pulicarus* was predicted in 2007 from transcriptomics [58]. It is a 16-residue peptide of the 4/6 disulfide subclass, and shares Loop I homology with AuIB, except for an additional *N*-terminal Gly residue (Table 1). Pu1.2 can inhibit HVA Ca^2+^ channels by 27% at 1 µM, but it is not active at α9α10 nAChR [59]. In 2016, Carstens and colleagues evaluated the activity of all topoisomers of Pu1.2 to gain insight into the structural requirements for Ca^2+^ channel inhibition via the GABA_B_ receptor [59]. All three isomers of Pu1.2 (native globular, ribbon and bead) were able to inhibit Ca^2+^ channels in rat DRG neurons with no significant difference in activity despite NMR spectroscopy revealing high structural diversity across the isomers. No disulfide shuffling was observed during the testing, leading the authors to attribute the observed activity to each specific isomer.

It is evident that there is still confusion over the mechanistic pathway that leads to analgesia invoked by α-conotoxins. Analgesia is most likely mediated by multiple pathways that still require full elucidation in different pain models. Results to date suggest that α-conotoxin analgesia could occur via different pain-signalling pathways depending on the tissue and the type of pain [60]. The main focus of research efforts is currently between two targets, the α9α10 nAChR and the GABA_B_ receptor. Conotoxin analogues that have selectivity for one over the other are highly desirable tools to dissect the mechanisms of analgesia. In order to design selective analogues, an in-depth understanding of the interaction with the target receptor and the structure-activity relationships is necessary. Although there have been numerous structural studies of α-conotoxins in an attempt to correlate structure to activity, a great deal of structural diversity and target diversity has made it difficult to achieve this. Available structural data relies on a combination of NMR spectroscopy [42,43,45,61], X-ray diffraction [62], pH titrations [63] and in silico studies [64]. In reality, these techniques may not unequivocally connect high-resolution solution structures to in vivo conformation and activity. Interaction of α-conotoxins with the numerous nAChR subtypes relies on interactions deep within the AChR receptor binding site. Hydrophobic patches are known to anchor the α-conotoxin within the acetylcholine binding site, with sub-type selectivity being provided via specific hydrogen bonding and salt bridge formation [65].

Fewer structural data exist for the more recently discovered inhibition of HVA Ca^2+^ channels via GABA_B_ receptor agonism. However, evidence is mounting that a peptidomimetic based on modification of a truncated Loop I sequence, a region of high homology across the α-conotoxin family, could provide a potent lead analgesic compound capable of pain modulation via direct interaction with dorsal root ganglion neurones [66]. A more complete understanding is required to progress these molecules as drug leads, and currently some of the best tools to probe these structures are peptidomimetics. Towards this end, the stabilising and flexible nature of the highly conserved disulfide bridges can be modulated to provide key information about the bioactive peptide topography and potentially provide analogues with optimised receptor binding, selectivity and potency.

## 3. Disulfide Replacement Strategies 

### 3.1. The Importance of the Disulfide Bridge 

It is clear that the secondary structure and exquisite biological specificity of α-conotoxins is modulated by their highly-conserved disulfide bridge framework. Disulfide bridges perform catalytic, structural and allosteric roles to modulate peptide activity. Catalytic disulfide bridges are directly involved in redox transformations that contribute to peptide bioactivity (i.e., receptor activation), whereas structural disulfide bonds are redox-inactive and provide conformational restraint and stabilise tertiary structure [67]. Allosteric disulfide linkages relay a biological function through conformational change at an external site, often arising from redox transformation [67,68,69]. In addition to this behaviour, it is important to recognise that disulfide bridges can be inherently unstable in biological environments. These bonds can be highly susceptible to reduction, thiol exchange and enzymes, which can have a significant influence on the structure of the peptide. For example, disulfide scrambling, resulting from thiol exchange, can lead to refolding of topoisomers with concomitant structural change and hence altered biological activity [70]. The globular isomer is widely regarded as the biologically active topoisomer for the majority of α-conotoxins. Folded disulfide-containing peptides typically have a stable structure, limiting access of the cleavage sites and conferring proteolytic stability compared to their linear or reduced counterparts [71,72]. As a result, disulfide reduction or scrambling destabilises the structure and provides greater opportunity for proteolytic degradation.

In an effort to identify the relationship between disulfide geometry and mechanism of action, S–S geometries are defined by the dihedral and torsional angles between the Cβ, Sγ and Sγ′ and Cβ′, Sγ′ and Sγ and the rotational angles of bonds χ1, χ2, χ3, χ1′ and χ2′ [67,68,69]. The torsional and dihedral angles have been shown to correlate with the functional assignment of the disulfide bonds as structural, catalytic, or allosteric. For example, allosteric disulfide bonds typically form a −RHStaple conformation across two antiparallel β-sheets that pucker to accommodate the strained geometry [68]. The increased strain contributes to susceptibility to redox transformation that may allow formation of the bioactive topography; this susceptibility is also reflected in the redox potentials of disulfide bridges: functional disulfide bridges range between −95 to −335 mV, whereas inert structural disulfides possess redox potentials as low as −470 mV [73]. It has also been hypothesised that the metabolic instability of the disulfide bridge is an evolutionary mechanism for modulating activation of the peptide structure, in essence a biological on/off switch [73,74].

**Figure 3:**
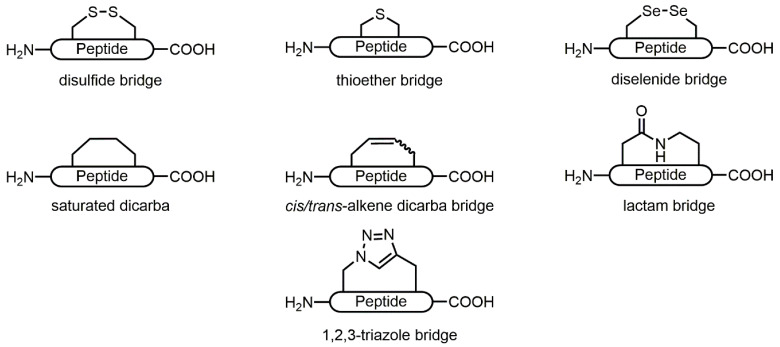
Disulfide mimetic strategies used in α-conotoxin sequences. Bridging cystine replacements include those generated from proteinaceous residues (diselenide, lactam bridges) and non-proteinaceous residues (thioether, hydrocarbon and triazole bridges).

Allosteric disulfide bridge modification that leads to a conformational shift may also generate the bioactive topography that provides optimal receptor binding. Difficulty arises in characterising the bioactive geometry of these disulfide bonds as they are invisible by ^1^H NMR spectroscopy. Reasonable conformational estimates can be made, however, based on the constraints of the neighbouring atoms [75]. Moreover, cystine bridges may be artificially represented in the solid state (i.e., X-ray crystallography) due to non-native contacts and conditions employed to obtain the crystals and structures. Thus, indirect assessment of bioactive geometry is generally inferred through in vitro and in vivo structure-activity relationships. It is therefore of interest to replicate bioactive topography via constrained bridges of well-defined and predictable geometry that are also metabolically stable. To date, cystine mimetics in α-conotoxins have been generated via diselenide, thioether, lactam, dicarba and 1,2,3-triazole bridges (Figure 3 and Table 2).

### 3.2. Diselenide Bridges

Selenocysteine is the 21st naturally-occurring proteogenic amino acid and structurally related to cysteine. The sidechain-bearing selenol has a significantly lower pKa of ~5.7 compared to the pKa of ~8.5 of cysteine’s thiol moiety, and consequently exists as the corresponding selenoate (R-Se^−^) at physiological pH [76]. Selenocysteine rapidly oxidises to form a diselenide bridge that is analogous to cysteine to cystine oxidative bridge formation.

Diselenide bridges are typically assembled in conotoxins using solid-phase peptide synthesis employing the Boc protecting strategy. This approach minimises reported racemisation and susceptibility towards piperidine-catalysed β-elimination of selenocysteine residues in the Fmoc strategy [77,78]. Diselenide bridge mimetics were first used in α-conotoxin ImI in 2006 [79]. ImI, from *Conus imperialis*, contains 12 amino acid residues with two disulfide bridges linking the backbone in a globular CysI-III, CysII-IV framework (Table 1, Figure 2) [80,81]. Interestingly, it is a selective inhibitor of the α7 nAChR but was found to be devoid of analgesic activity when tested in a warm water tail withdrawal assay [82]. Diselenide substitution in either Loop I or Loop II, or replacement of both, provides only a marginal increase in potency at the α7 nAChR, which is attributed to increased hydrophobic interactions between the diselenide bridge and the receptor [79]. Several diselenide-containing α-conotoxins (MI, BuIA, AuIB, Vc1.1 and PnIA) were prepared, and displayed similar or improved nAChR activity to the native sequences [83]. A notable improvement in potency was observed for the Loop I diselenide analogue of MI (IC_50_ = 9 nM) compared to the native peptide (IC_50_ = 26 nM) at (α1)_2_β1δγ nAChR. A diselenide Loop I analogue of AuIB also displayed significant improvement in potency (IC_50_ = 260 nM) compared to the native sequence (IC_50_ = 3100 nM) at the α3β4 nAChR. Interestingly, for each of these diselenide α-conotoxin analogues, ^1^H NMR and CD spectroscopy revealed that the improvements in activity were not accompanied by gross structural deviation from the native three-dimensional structure [79,83]. The observed activity was therefore attributed to the increased hydrophobicity of the selenium, as reported for the ImI analogue [83,84]. No functional data have been reported for diselenide α-conotoxin analogues on GABA_B_ receptors.

Diselenide bridges form more rapidly than disulfide bridges, allowing for regioselective folding of α-conotoxins to the globular isomer in near quantitative yields [79,83,85,86,87]. This concept has been further expanded into conotoxins containing three disulfides, MrVIB and SIIIA, where a single diselenide bridge replacement directed folding to preferentially give the native framework [88,89].

### 3.3. Triazole

1,2,3-Triazoles have been utilised in chemistry for a range of purposes owing to their excellent proteolytic stability and orthogonality to most other naturally-occurring functionalities [90]. In addition to replacement of amide bonds, they have been used as stable and rigid disulfide surrogates [90,91,92,93]. Triazole motifs are generated using well-established copper-catalysed azide-alkyne cycloaddition (CuAAC) from propargylglycine and azidoalanine residues [94,95]. The first use of a 1,4-disubstituted-1,2,3-triazole linkage in conotoxins was reported in 2015 to generate MrIA χ-conotoxin analogues, where the triazole direction and flexibility were found to impact inhibition of norepinephrine transportation down to micromolar concentrations [95]. Formation of the triazole linkage via azidoalanine and alkynyl residues in positions 4 and 13, respectively, was more potent (IC_50_ = 1.73 µM) than its corresponding regioisomer (IC_50_ = 3.84 µM). However, the 1,5-disubstituted-1,2,3-triazole has been suggested as a more effective disulfide mimic due to its orientation and geometry [96]. The 1,5-disubstituted isomer can be obtained via ruthenium(II)-catalysed azide-alkyne cycloaddition (RuAAC) [97]. The only reported triazole disulfide replacement in α-conotoxins was achieved in GI, where RuAAC was used to generate the 1,5-disubstituted-1,2,3-triazole replacement of the Loop I or Loop II bridge [98]. GI is a 13 residue α-conotoxin from the venom of *Conus geographus* that acts as a competitive antagonist for the muscular nAChR with excellent subtype specificity [99,100]. In vitro assessment at human nAChR indicated that the Loop I triazole analogue was devoid of activity while the Loop II triazole was slightly more active than native GI [98]. Whilst ^1^H NMR spectroscopy was not used to assess the structures of these analogues, it is possible that the steric bulk of the triazole motif may disrupt the binding domain of the Loop I sequence, disrupt the conserved Loop I β-turn peptide structure through restricted flexibility and orientation, or fail to provide key disulfide receptor interactions. Furthermore, replacement of sulfur may limit the hydrophobic interactions purported to be important for nAChR interactions [79,83,84]. Nevertheless, triazole replacement led to a 10-fold increase in plasma stability. To the best of our knowledge, experiments regarding triazole replacements in GABA_B_ active α-conotoxins have not been reported.

**Table 2:** Effects of disulfide replacement on α-conotoxin sequences.

Replacement Strategy	Conotoxin (Targeted Bridge)	Primary Receptor Target	Improved In Vitro Activity?	Retained Native Structure?	Reduced Disulfide Scrambling?	Improved Plasma Stability?	Ref
Diselenide	MI (3–13)	(α1)_2_β1δγ nAChR	✓	n.r.	n.r.	n.r.	[83]
	AuIB (2–8)	α3β4 nAChR	✓	✓ ^a,b^	✓	≈	[83]
	AuIB (3–15)	α3β4 nAChR	✓	✓ ^a,b^	✓	≈	[83]
	ImI (2–8)	α7 nAChR	✓	✓ ^a,c^	✓	✓	[79,83]
	ImI (3–12)	α7 nAChR	✓	✓ ^a,c^	✓	✓	[79,83]
	ImI (2–8,3–12)	α7 nAChR	✓	✓ ^a,c^	✓	✓	[79]
	Vc1.1 (2–8)	α3β4 nAChR	✓	n.r.	n.r.	n.r.	[83]
	(A10L)-PnIA (2–8)	α7 nAChR	≈	n.r.	n.r.	n.r.	[83]
	(A10L)-PnIA (3–16)	α7 nAChR	n.r.	✓ ^d^	n.r.	n.r.	[83]
Triazole	GI (2–7)	muscle nAChR	✗	n.r.	✓ ^i^	n.r.	[98]
	GI (3–16)	muscle nAChR	✓	n.r.	✓ ^i^	n.r.	[98]
Thioether	GI (2–7,3–16)	muscle nAChR	**✗**	n.r.	✓ ^i^	n.r.	[101]
	ImI (2–8)	α7 nAChR	✗	✓ ^c^	✓ ^i^	n.r.	[102]
	ImI (3–12)	α7 nAChR	≈	✓ ^c^	✓ ^i^	n.r.	[102]
	ImI (2–8,3–12)	α7 nAChR	✗	✓ ^b^	✓ ^i^	n.r.	[102]
Lactam	(*des*-Glu1)-GI (2–7)	undefined	✗	n.r.	✓ ^i^	n.r	[103]
	(*des*-Glu1)-GI (3–16)	undefined	≈	n.r.	✓ ^i^	n.r.	[103]
	SI (2–7) ^e^	α_2_βγδ nAChR	✗	n.r.	✓ ^i^	n.r.	[104]
	SI (2–7) ^f^	α_2_βγδ nAChR	✗	n.r.	✓ ^i^	n.r.	[104]
	SI (3–13) ^e^	α_2_βγδ nAChR	✓	n.r.	✓ ^i^	n.r.	[104]
	SI (3–13) ^f^	α_2_βγδ nAChR	✗	n.r.	✓ ^i^	n.r.	[104]
Dicarba	ImI *cis*-(2–8)	α7 nAChR	≈	✓ ^c^	✓ ^i^	n.r.	[105]
	ImI *trans*-(2–8)	α7 nAChR	✗	✓ ^c^	✓ ^i^	n.r.	[105]
	Vc1.1 *cis*-(2–8)	GABA_B_	✓ ^g^	✓ ^c^	✓ ^i^	n.r.	[84]
	Vc1.1 *trans*-(2–8)	GABA_B_	✓ ^g^	✗ ^c^	✓ ^i^	n.r.	[84]
	Vc1.1 *cis*-(3–16)	GABA_B_	✗ ^h^	✓ ^b^	✓ ^i^	n.r.	[84]
	Vc1.1 *trans*-(3–16)	GABA_B_	✗ ^h^	✓ ^c^	✓ ^i^	n.r.	[84]
	RgIA *cis*-(2–8)	GABA_B_	✓ ^g^	✓ ^c^	✓ ^i^	✓	[106]
	RgIA *trans*-(2–8)	GABA_B_	✓ ^g^	✗ ^b^	✓ ^i^	✓ ^i^	[106]
	RgIA *cis*-(3–16)	GABA_B_	✗ ^h^	✓ ^c^	✓ ^i^	✓ ^i^	[106]
	RgIA *trans*-(3–16)	GABA_B_	✗ ^h^	✓ ^c^	✓ ^i^	✓ ^i^	[106]

a: Determined by CD analysis; b: determined by secondary NMR chemical shift analysis; c: full NMR structural determination (3D-structure); d: X-ray analysis; e: Glu/Lys; f: Lys/Glu; g: GABA_B_ active, inactive at α9α10 nAChR; h: α9α10 nAChR active, inactive at GABA_B_; i: determined by inference; ≈: equivalent to parent sequence; n.r.=not reported.

### 3.4. Thioether

Thioether bridges have been used extensively as non-reducible surrogates of disulfide bridges and have been introduced in a large variety of natural cyclic peptides to extend their activity and in vivo stability [107]. Examples include oxytocin [108,109,110,111], calcitonin [112], compstatin [113] and enkephalin [114,115]. Thioether bridges are redox-stable isosteres of the cystine linkage where one of the sulfur atoms is replaced with a methylene group. They are hypothesised to closely mimic the geometry of the disulfide without inducing structural perturbation that may result from introduction of steric bulk, i.e., triazole [102]. They are typically introduced via cyclisation of a cysteine thiol with a γ-chlorinated side chain [107,116], an approach that has been performed on solid support to generate the thioether analogues of α-conotoxin GI and ImI [101,102]. Thioether replacement of Loop I or Loop II in GI generated two isomers that were resolved by HPLC, but each isomer contained a mixture of several conformers that were found to interconvert on the NMR timescale [101]. Both isomers were evaluated for biological activity in muscle nAChRs and were found to be more than 244-fold less potent than the native peptide.

Regioselective thioether replacement to generate stable mimetics of Loop I and Loop II ImI analogues showed only a single conformation by ^1^H NMR spectroscopy with conserved backbone chemical shifts [102]. In vitro assessment against the α7 human nAChR showed that Loop II thioether ImI retained native activity (IC_50_: 0.38 µM vs. 0.38 µM for native ImI). However, both Loop I thioether ImI and double Loop I and Loop II thioether ImI analogues showed a 3-fold reduction in activity at the α7 nAChR sub-type (IC_50_: 1.09 µM and 1.28 µM, respectively). Once again, this may reflect the loss of key hydrophobic S–S interactions with the nAChR given the conservation of native backbone structure. Despite the successful mimicry of the disulfide bond, introduction of thioether bridges poses a greater synthetic challenge relative to other replacement strategies due to the propensity for β-elimination, racemisation and lengthy synthetic protocols.

### 3.5. Lactam Bridge

Lactam bridges have been extensively used for the stabilisation of α-helices or β-turns in peptides [117,118,119]. In addition, this motif has been used as a substitute for a disulfide bridge in several biologically-relevant peptides such as human urotensin II [120], endothelin-1 [121], angiopeptin (an analogue of somatostatin) [122], an immunodominant epitope of the HIV virus [123], gomesin [71] and oxytocin [108]. Lactam bridge insertion has been reported for α-conotoxins GI and SI.

Two lactam analogues of (*des*-Glu1)-GI (which possesses native GI activity [124]) were synthesised via side-chain condensation of Asp and a truncated Lys derivative ((2*S*)-2,3-diaminopropanoic acid) [103]. Replacement of the Loop I disulfide bridge resulted in a 154-fold loss of activity compared with the (*des*-Glu1)-GI sequence, whereas the Loop II replacement retained native activity. The loss of activity observed for the Loop I amide analogue may arise from an altered hydrogen bonding network induced by the amide motif and subsequent inability to create the receptor-binding conformation. Alternatively, altered bridging geometry could impede receptor interaction or the replaced disulfide bridge in Loop I of (*des*-Glu1)-GI may be directly involved in binding to the receptor [103].

Disulfide replacement with lactam functionality was also conducted on α-conotoxin SI, a 13-residue peptide found in the venom of *Conus striatus* and active at the muscle α/δ subunit of nAChRs [104,125]. Individual replacement of the two disulfide bridges and opposing orientations of the lactam bridge (Glu/Lys or Lys/Glu) generated four analogues. Analogous to (*des*-Glu1)-GI, the lactam analogues of SI also displayed very different activities at the nAChR: both Loop I lactam SI analogues were inactive, but Loop II lactam analogues showed highly divergent activity dependent on the orientation of the lactam bridge. The Loop II [Lys^3^, Glu^13^]-analogue was ~60-fold less potent than native SI but the Loop II [Glu^3^, Lys^13^]-analogue was ~70-fold more potent; a striking 4000-fold difference [104]. In SI, the Loop I disulfide is embedded within the central hydrophobic core of the tertiary structure [126]. The flexibility that arises from the additional methylene groups of the precursor Lys and Glu residues compared to the native disulfide likely contributes to the loss of native tertiary structure in the Loop I analogues through a less defined tertiary architecture [126]. The additional loss of sulfur-based hydrophobic interactions with the receptor may also contribute to this lack of activity. The Loop II disulfide bridge is remotely positioned from the bulk of the conotoxin structure [126], and the longer bridge and its incumbent flexibility is well tolerated in this region. The striking structure-activity divergence observed in the two regioisomers may result from additional or advantageous reconfiguration of hydrogen bonding induced by the amide bridge. Additional ^1^H NMR spectroscopy would provide insight into the structural constraints imposed on the tertiary structure by the lactam bridge. It was hypothesised that the Loop II disulfide of both SI and GI provides structural support to the peptide conformation, whereas Loop I actively participates in receptor interactions at nAChRs, hence the loss of activity upon its replacement [104].

### 3.6. Dicarba Bridges

Dicarba bridges are carbon-based isosteres of the disulfide bridge. The atom replacement is conservative, and does not introduce an artificial dipole (C and S are isoelectronic) or steric bulk to the bridge site. Dicarba bridges provide an elegant approach to probing the bioactive topography of the disulfide bridge within a peptide through defined hybridisation and stereochemistry. The dihedral and torsional angles can be tuned through varying hybridisation of the carbon-based bridges to optimise and promote receptor binding and therefore improve therapeutic value. Moreover, dicarba bridges are enzymatically non-reducible under physiological conditions and therefore provide improved plasma stability.

Saturated dicarba bridge mimetics were initially installed in peptide sequences using orthogonally-protected 2,7-diaminosuberic acid residues [127,128,129]. The use of olefin metathesis and hydrogenation to achieve the same end has more recently been facilitated through the development of functional-group-tolerant Grubbs ruthenium(II)-alkylidene catalysts. Application of this catalytic approach generates unsaturated (olefinic) dicarba bridges using two sequence-installed non-proteinaceous allylglycine residues [130,131]. Ring closing metathesis in peptides has been reviewed recently [132].

α-Conotoxin ImI was the first conotoxin to undergo dicarba replacement in 2009 [105]. The Loop I [2,3,4,5,6,7,8]-dicarba bridge in ImI generated both the *cis* and *trans* C=C geometric isomers. Remarkably, the *cis*-dicarba-ImI possessed comparable activity to native ImI at rat α7 nAChR in the presence of acetylcholine, showing 60% and 69% inhibition, respectively. Conversely, the *trans*-dicarba-ImI was inactive. Structural analysis by NMR spectroscopy indicated that the backbone structures deviated from the native form at the site of mutation. Correspondingly, Loop II showed a noticeable shift in orientation with respect to the rest of the peptide, and the *N*-terminal region was twisted due to disruption of native Gly1–Ser4 hydrogen bonding [105]. Importantly, the known nAChR receptor binding residues Asp5–Arg7 had well conserved conformations in both the dicarba and native forms [105,133]. It is therefore likely that the nAChR subtype affinity between the *cis* and *trans* isomers is dictated via receptor interactions with other residues.

A similar disulfide replacement strategy was employed in α-conotoxins Rg1A and Vc1.1. In 2013, selective replacement of both the Loop I and Loop II disulfide bridges in Vc1.1 with an alkene bridge was performed to generate four analogues—*cis* and *trans* Loop I dicarba Vc1.1 and Loop II dicarba Vc1.1 [84]. Remarkably, Loop I dicarba Vc1.1 displayed potent agonism of the GABA_B_ receptor but complete loss of activity at the α9α10 nAChR. In stark contrast, the Loop II dicarba Vc1.1 was potently active at the α9α10 nAChR but had completely lost activity at the GABA_B_ receptor. The discovery of this structure-activity relationship delineated functional regions of the peptide and provided valuable tools for exploring the mechanism of analgesia. Interestingly, the backbone conformation of *cis* Loop I dicarba Vc1.1 and both *cis* and *trans* Loop II dicarba Vc1.1 were comparable to native Vc1.1. However, the *trans* Loop I dicarba Vc1.1 demonstrated significant perturbation of the tertiary structure, and its corresponding loss of activity was not surprising. Given the similarity of three analogues, it was suggested that the difference in activity resulted from the cysteine residues themselves. Replacement of the sulfur with inert carbon was postulated to lead to a loss of disulfide exchange with the nAChR; this hypothesis matched the observed biological activity and is also consistent with reported nor-Loop I disulfide studies [79,103,104]. Molecular dynamics simulations of the ligand-binding domain of α9α10 nAChR complexed with the native Vc1.1 and dicarba analogues showed a loss of contact between the alkene carbons of *cis* Loop I dicarba Vc1.1 and key residues of the binding pocket [84]. Furthermore, a loss of a stacking interaction with a disulfide bond on the principal face of the binding site was also evident in the models, which could explain its loss of activity at the nAChR.

The stability of these dicarba analogues in human serum increased the lifetime of the peptide through minimising disulfide scrambling and proteolysis. For example, the *cis* isomer of Loop I dicarba RgIA showed increased stability, with 30% of the peptide still detected after 1 h compared to a half-life of 3 min for the native Rg1A sequence [106,134].

### 3.7. Disulfide-Based Target Tunability

To examine the influence of loop regions on HVA Ca^2+^ channel inhibition, truncated peptide sequences of Vc1.1 and Pu1.2 were examined in vitro [59]. Interestingly, Loop I truncated [1,2,3,4,5,6,7,8,9]-Pu1.2 retained inhibition of HVA Ca^2+^ channels via GABA_B_ agonism akin to the native peptide, whereas Loop II truncated [9,10,11,12,13,14,15,16]-Pu1.2 was inactive. Analogous truncation to give the Loop I [1,2,3,4,5,6,7,8]-Vc1.1 analogue resulted in comparable inhibition at the GABA_B_ receptor compared to the native sequence. Interestingly, Vc1.1, RgIA, EpI and ImI all share sequence homology in the Loop I 1-8 sequence but vary in size and residue complexity in Loop II. Whilst GABA_B_ receptor activity appears to be primarily modulated via Loop I, the difference in target selectivity of ImI, and lack of in vivo analgesia, indicates that Loop II can play a key role in modulating binding affinity and activity at the GABA_B_ receptor. Moreover, previously-described analgesic Loop I dicarba Vc1.1 and RgIA analogues were selective for the GABA_B_ receptor and devoid of α9α10 nAChR activity, further suggesting that a Loop I-contained epitope is solely capable of providing potent in vivo analgesia [84,106]. Consequently, the remainder of this review focuses on the impact of the *N*-terminal residues defining Loop I and highlights their engagement to provide agonism of GABA_B_ receptors as a primary pathway to analgesia.

## 4. Loop I Residue Analysis

### 4.1. N-Terminus

The *N*-terminal ammonium group does not appear to play a crucial role in the analgesic activity of α-conotoxins. Acetylation of the [N9R]-Vc1.1 analogue resulted in a reduction of analgesic activity in partial sciatic nerve ligation (PNL) rats compared to the [N9R]-Vc1.1 sequence, whereas benzoylation of [N9R]-Vc1.1 displayed a greater potency in HEK293T cell co-expressing GABA_B_ and Ca_V_2.2 channels [133,135]. Backbone-cyclised Vc1.1 also displayed an improved inhibition of HVA Ca^2+^ channels, further indicating that a free *N*-terminal amine is unnecessary for biological activity [38]. Conversely, our in-house data revealed that fluoropropionyl ligation to the *N*-terminal of Vc1.1 leads to loss of in vivo activity (unpublished). Given the reported tolerance for both acetylation and benzoylation of the Vc1.1 *N*-terminus, additional hydrogen bonding to the fluorine may disrupt the tertiary structure, resulting in reduced receptor activity. There have been no other reported influences of *N*-terminal modifications in analgesic α-conotoxins at nAChR or GABA_B_ receptors.

### 4.2. Gly1

Glycine is well conserved as the *N*-terminal residue in most α-conotoxins. Additional *N*-terminal Gly residues, as well as Pro and Gln residues, have been observed in a few sequences, e.g., Pu1.2, Kn1.2, Tx1.2 and AnIB (Table 1). Deletion of the *N*-terminal Gly residue has only been reported for α-conotoxin AnIB, which has not been tested for in vivo analgesia [136]. The sequential deletion of one or both Gly residues at the *N*-terminus influenced the dissociation constant of AnIB analogues at α3β2 nAChR. The shorter analogue (*des*-Gly1-*des*-Gly2)-AnIB showed an increased dissociation constant, which resulted in a 20-fold reduced potency [136]. This reduced activity does not correlate with α-conotoxin [*des*-1-4]-GID analogue, which showed no change at the α3β2 nAChR compared to the full sequence but retained a similar residue 4–7 Loop I sequence to AnIB and other α-conotoxins [137]. The difference is therefore likely due to the influence of the Loop II sequence defining receptor subtype specificity. These sequences have not been examined for activity at the GABA_B_ receptor.

On HVA Ca^2+^ channels, the replacement of Gly1 in Vc1.1 with neutral Ala or negatively-charged Asp did not affect inhibitory activity [138]. However, positively-charged Lys substitution resulted in loss of activity. Mutation at the Gly1 position with these three residues was well tolerated at α9α10 nAChR, where biological activity was retained. Furthermore, deletion of Gly1 in [N9R]-Vc1.1 resulted in reduced analgesia compared to [N9R]-Vc1.1, but comparable activity to native Vc1.1 in in vivo pain models [135].

### 4.3. Cys2 (and Cys8)

Discussed previously in Section 3.

### 4.4. Cys3

Cys3 is typically not modified in structure-activity relationship studies as it is a crucial residue in the Loop II disulfide bridge. In order to avoid disulfide isomerisation in the truncated Loop I Vc1.1 sequence, it is replaced with Ser without impacting activity; comparable inhibition of rat HVA Ca^2+^ channels was noted by the authors for [1,2,3,4,5,6,7,8]-Vc1.1, [1,2,3,4,5,6,7,8]-[C3S]-Vc1.1 and native Vc1.1 at 1 µM [59]. Replacement of Cys3 with the linear alkynyl-derived residue in the linear triazole GI precursor showed significantly reduced activity at nAChRs (IC_50_ = 203 nM vs. native IC_50_ = 9.8 nM) [98]. This supports once again that the Loop II sequence plays a role in nAChR activity but is not solely responsible for activity at this target. No further modifications have been reported beyond disulfide replacement strategies.

### 4.5. Ser4

Ser4 is well conserved across numerous α-conotoxin sequences (Table 1), and the importance of Ser4 substitution has been analysed in several α-conotoxin analogues. The S4A analogues of RgIA, PeIA and AuIB showed no significant change in activity at α9α10, α3β2 and α3β4 nAChR, respectively [43,139,140], but this substitution reduced activity at the α3α2 and α6/α3β2β3 nAChR subtypes for MII [141,142]. A marginal increase in activity was observed for the [S4A]-BuIA analogue on α6/α3β2β3 nAChRs [143].

Point mutations of Ser4 in Vc1.1 to the positively-charged residues Lys or Arg led to increased potency at the α9α10 nAChR, whereas replacement with neutral Ala or negatively-charged Asp resulted in decreased activity [138,144]. This correlates with computer modelling studies that show Ser4 participating in hydrogen bonding with the α9α10 nAChR [145]. In an effort to strengthen this hydrogen bonding interaction, replacement of Ser4 with diaminobutyric acid (which has a greater electrostatic attraction for hydrogen bonding) produced a significant increase in α9α10 nAChR inhibition [145]. However, analogous replacement with Lys resulted in a slight loss of inhibitory activity, suggesting an optimal length for hydrogen bonding with the receptor. The impact of these mutations appears to arise from enhancement or destruction of hydrogen bonding with the receptor.

Structure-activity relationship data for this residue at HVA Ca^2+^ channels are under-represented in the literature. Ser4 substitution in Vc1.1 with Lys or Asp led to maintenance of the inhibitory effect at HVA Ca^2+^ channels [138]. However, replacement with non-polar Ala led to a significant reduction in potency at these channels. Ser4 substitution to Thr in α-conotoxin RgIA, in combination with several other point mutations, generated RgIA4, an analogue devoid of GABA_B_ activity but suitably enhanced for activity at nAChR receptors [15]. Importantly, the RgIA4 analogue produced oxaliplatin-induced cold allodynia akin to its parent peptide. The isolated effect of the S4T substitution, however, cannot be determined from this study.

### 4.6. Asp5

Asp5 is one of the key three residues (Asp-Pro-Arg) that has been identified as key to binding with α9α10 nAChRs [43,144]. Consequently, there are few reported modifications to this residue. Substitution to Ala or Lys leads to a loss in activity at both the α9α10 nAChR and HVA Ca^2+^ channels receptors in Vc1.1 [138,144]. Similarly, RgIA mutation to Glu5 [15] and D-Asp5 [134] both result in loss of potency at the α9α10 nAChR, highlighting the importance of side chain length and stereochemistry at this position. There has been no reported modification to this residue that leads to retention or increase of biological activity.

### 4.7. Pro6

Along with the CysI-III, II-IV disulfide arrangement that characterises the α-conotoxin family, Pro6 appears to be an archetypal structural feature. Appearing at this exact position in all known examples throughout this family apart from the lesser known α-conotoxins Lp1.1, ImII and LtIA [146], this residue is thought to induce the 3_10_ helical structure common to α-conotoxins [147]. In combination, various point mutation and structural studies suggest that the helix domain is non-essential, but modification of Pro6 in any capacity results in an inactive sequence [133,142,148,149,150,151].

Post-transcriptionally-modified analogues of Vc1.1 contain (4*R*)-hydroxyproline at position 6 ([P6O]-Vc1.1). The effect of (4*R*)-hydroxylation is well documented on the proline ring, stabilising the *trans* X_AA_-Pro state through inductive effects [152,153]. The effect of (4*R*)-hydroxylation has been extensively explored in stabilising collagen [154,155], but its effect within conotoxin sequences is yet to be fully investigated. It is understood that the native Vc1.1 sequence and (4*R*)-hydroxylated analogues are structurally analogous but the hydroxylated analogue produces no in vivo analgesia in nerve injury or neuropathic pain models [156]. Incorporation of a *cis*-directing proline analogue (e.g., (4*S*)-hydroxyproline) is known to greatly disrupt the gross structure but functional data for this analogue have not been reported [157].

Following the clinical trial of Vc1.1, subsequent investigation identified that [P6O]-Vc1.1 retained α9α10 nAChR inhibition comparable to Vc1.1 [17]. Modelling of [P6O]-Vc1.1 in the α9α10 nAChR binding site suggested a complimentary interaction between the polar hydroxyproline residue at position 6 with a receptor Asp residue [156]. Significantly however, this analogue showed no mechanical allodynia. While Vc1.1 potently inhibits HVA Ca^2+^ channel currents (IC_50_ = 1.7 nM (rat)), [P6O]-Vc1.1 displayed no inhibition at the same target. Furthermore, substitution of Pro6 for Ala, Asp or Lys also results in a loss of in vitro activity at both α9α10 nAChR and HVA Ca^2+^ channels [138]. NMR spectroscopy of proline substitutions has shown complete loss of secondary structure [144], and so these results are not surprising. Together these results provide strong support for GABA_B_ receptor modulation as a primary pathway to analgesia [17].

Stromgaard and coworkers targeted Pro6 in α-conotoxins ImI and [A10L]-PnIA and explored the corresponding antagonistic activities at the α7 nAChR receptor [158]. Interestingly, the addition of a (5*R*)-phenyl group stabilised receptor interactions and decreased IC_50_ values by ~5 fold in ImI [158]. However, the addition of (2*S*,4*S*)- and (2*S*,4*R*)-fluoroproline surrogates, which direct to *cis* and *trans* Xaa-Pro states, respectively, both saw a decrease in receptor affinity. Unfortunately, in vivo analgesic data were not reported. Documented cases outside the α-conotoxin family exist where *cis-trans* isomerisation plays a critical role in forming the bioactive conformation. For example, the bioactive type VI β-turn in δ-conotoxin EVIA was stabilised through the addition of a *cis*-directing pseudoproline [159].

### 4.8. Arg7

In the Vc1.1 sequence, Arg7 is within the highly-conserved triplet sequence Asp-Pro-Arg, required for high-affinity binding to both GABA_B_ and α9α10 nAChR [138]. A loss of activity was observed with Ala and Asp replacement [138,144]. Even substitution with a positively-charged Lys caused a reduction in affinity for both receptors [138,144]. The same was observed for the [R7K]-RgIA analogue at α9α10 nAChR [144]. This highly-conserved region is found in ImI and associated with binding to the α7 nAChR subtype [160]. Substitution of Arg7 in ImI with Lys, Glu and Gln resulted in a loss of activity at α7 receptors [133]. A truncated analogue of Vc1.1, [1,2,3,4,5,6,7,8]-Vc1.1, was also able to inhibit the α7 nAChR receptor at 1 µM concentration [59]. Hence, the Loop 1 Arg residue appears to be key for all receptor interactions for Vc1.1 and other structurally-related Loop I analogues (e.g., RgIA, ImI).

## 5. Concluding Remarks

Approximately 70 α-conotoxin sequences have been reported in the literature and more than half of these peptides share a highly-conserved *N*-terminal sequence tethered by a cystine bridge. Eight out of 67 of these peptides share a common Loop I sequence of GCCSDPRC; two are GABA_B_ active and analgesic (Vc1.1 and Rg1A), three have no reported GABA_B_ activity and possess no analgesic activity (Vc1a, ImI, EpI), two possess a 4*R*-hydroxyproline residue and are likely to be inactive (Reg 1b and 1c), and details about Reg1d are unknown. Despite the Loop I sequence homology, this variation in activity profile highlights the biological significance of Loop II residues, which show high variability in structure and number across the α-conotoxin family (Table 1). This sequence variation is responsible for the exquisite sub-type selectivity observed at mammalian nAChRs. Stabilisation of α-conotoxin tertiary structure is facilitated via the rich array of polar residues scattered throughout these compact peptides and indeed creation of the bioactive conformation appears to rely on accessing an optimised structure through a network of key hydrogen bonds and ionic interactions within the peptide itself and with the target.

This review has focussed on analgesic α-conotoxins that indirectly inhibit high-voltage activated (HVA) Ca^2+^ channels via agonism of GABA_B_ G protein-coupled receptors. Invariant Loop I cysteine and proline residues clearly play an important role in facilitating generation of the bioactive conformation at the GABA_B_ receptor. Dicarba replacement of the Cys2–Cys8 bridge results in variable GABA_B_ activity depending on bridge hybridisation and geometry, supporting the notion that the Loop I disulfide may play an allosteric role in receptor activation. Given the proximity of the invariant proline residue to the Loop I disulfide and key residues, it is possible that the puckering of the proline ring between Cγ-endo and Cγ-exo states, rather than *cis-trans* isomerization, may facilitate allosteric control of peptide conformation.

While some residues may favour the bioactive conformation, the above-described structure-activity review suggests that Loop II residues can strongly influence hydrogen bonding and ionic interactions through Loop I without significantly affecting backbone topography. Indeed, removal of Loop II residues in the form of truncated Vc1.1 analogues has been shown to provide analgesic peptides with GABA_B_ receptor activity. α-Conotoxin Vc1.1, Rg1A and ImI all share a common Loop I sequence but ImI is not analgesic. Both Rg1A and ImI are 4/3 subtype α-conotoxins, yet only Rg1A is highly susceptible to Loop II proteolytic degradation. Vc1.1, on the other hand, is a 4/7 subtype α-conotoxin and, like ImI, is highly resistant to proteolysis in plasma. Formation of the required Loop I topography for receptor activation might therefore be facilitated through in vivo Loop II cleavage, its reactive displacement within the receptor site, or simply the absence of deleterious Loop II interference as dictated by the primary sequence.

It is clear that conformationally-constrained analogues of the α-conotoxins have much to offer in elucidating the structure-activity relationships and receptor specificity of this important class of venom-derived peptides. The structural changes imposed by such conformational restraints are not always predictable, but solution NMR studies offer an efficient and effective means of defining those changes. In addition to serving as valuable molecular probes, conformationally-constrained analogues also show promise as therapeutic leads in the development of non-opioid analgesics.

## Figures and Tables

**Table 1:** Selected α-conotoxin sequences showing the conserved disulfide framework.

Conotoxin	Sequence																	Analgesia		UniProt ID	
			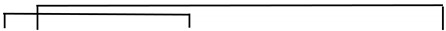																		
AnIB	G	G	C	C	S	H	P	A	C	A	A	N	N	Q	D	*Y*	C	*		n.r.	P0C1V7
AuIB	-	G	C	C	S	Y	P	P	C	F	A	T	N	P	D	-	C	*		✓	P56640
BuIA	-	G	C	C	S	T	P	P	C	A	V	L	Y	-	-	-	C	*		✓	P69657
EpI	-	G	C	C	S	D	P	R	C	N	M	N	N	P	D	*Y*	C	*		n.r.	P56638
GI	-	E	C	C	N	-	P	A	C	G	R	H	Y	S	-	-	C	*		✓	P01519
ImI	-	G	C	C	S	D	P	R	C	A	W	R	-	-	-	-	C	*		✗	P50983
Kn1.2	P	G	C	C	N	N	P	A	C	V	K	H	R	-	-	-	C	G		n.r.	D4HRK7
MI	G	R	C	C	H	-	P	A	C	G	K	N	Y	S	-	-	C	*		✓	P01521
MII	-	G	C	C	S	N	P	V	C	H	L	E	H	S	N	L	C	*		✓	P56636
MrI.I	-	G	C	C	S	H	P	A	C	S	V	N	N	P	D	I	C	*		✓	Q6PTD1
PeIA	-	G	C	C	S	H	P	A	C	S	V	N	H	P	E	L	C	*		✓	Q1L777
[A10L]-PnIA	-	G	C	C	S	L	P	P	C	A	L	N	N	P	D	*Y*	C	*		n.r.	P50984
Pu1.2	G	G	C	C	S	Y	P	P	C	I	A	N	N	P	L	-	C	*		✓	A1X8D8
Reg1d	-	G	C	C	S	D	P	R	C	K	H	E	-	-	-	-	C	*		n.r.	P85010
RgIA	-	G	C	C	S	D	P	R	C	R	Y	R	-	-	-	-	C	R		✓	P0C1D0
SI	-	I	C	C	N	-	P	A	C	G	P	K	Y	S	-	-	C	*		n.r.	P15471
Tx1.2	P	Q	C	C	S	H	P	A	C	N	V	D	H	P	E	I	C	*		n.r.	P0DPL9
Vc1.1	-	G	C	C	S	D	P	R	C	N	Y	D	H	P	E	I	C	*		✓	P69747

Highlighted: invariant cysteine residues, yellow; Loop I proline residues, blue; and serine residues, green. * = C-terminal amide, *Y* = sulfonated tyrosine, n.r. = not reported.
